# Pro-inflammatory cytokines disrupt *in vitro* preantral follicle development by targeting granulosa and theca cell functions

**DOI:** 10.3389/fendo.2025.1667019

**Published:** 2026-01-16

**Authors:** Aya Shirafuji, Makoto Orisaka, Natsumi Shimizu-Mizuno, Miki Uesaka, Yuko Fujita, Katsutoshi Mizuno, Masayuki Fujita, Yumiko Miyazaki, Toshimichi Onuma, Hideaki Tsuyoshi, Tetsuya Mizutani, Yoshio Yoshida

**Affiliations:** 1 Department of Obstetrics and Gynecology, Faculty of Medical Sciences, University of Fukui, Fukui, Japan; 2 Department of Cell Biology and Biochemistry, Division of Medicine, Faculty of Medical Sciences, University of Fukui, Fukui, Japan; 3 Department of Nursing, Faculty of Nursing and Welfare Sciences, Fukui Prefectural University, Fukui, Japan

**Keywords:** chronic inflammation, granulosa cell, oxidative stress, preantral follicle development, proinflammatory cytokine, theca cell, tissue fibrosis

## Abstract

**Background:**

Chronic low-grade inflammation is increasingly recognized as a contributing factor to female infertility. Elevated levels of pro-inflammatory cytokines such as TNF-α, IL-1β, and IL-6 are observed in ovarian conditions including obesity, polycystic ovary syndrome, endometriosis, and reproductive aging. However, how these cytokines affect early follicular development remains poorly understood. This study aimed to elucidate the effects of TNF-α, IL-1β, and IL-6 on granulosa cell (GC) and theca cell (TC) function during the preantral-to-antral transition—a critical checkpoint that determines follicular growth or atresia.

**Methods:**

We employed a three-dimensional culture model using mechanically isolated rat preantral follicles. Follicles were treated with TNF-α, IL-1β, or IL-6 in the presence or absence of FSH. We assessed follicular growth, steroid production, gene expression, reactive oxygen species accumulation, and type III collagen deposition using ELISA, quantitative PCR, and immunofluorescence analyses.

**Results:**

In the absence of FSH, none of the cytokines altered GC volume; however, TNF-α significantly suppressed FSH-induced GC proliferation. All three cytokines—TNF-α, IL-1β, and IL-6—increased TC volume without enhancing androgen production. FSH-stimulated estradiol and testosterone synthesis was markedly impaired, accompanied by downregulation of key steroidogenic genes including *Fshr*, *Cyp19a1*, *Lhcgr*, *Cyp11a1*, and *Cyp17a1*. TNF-α and IL-1β elevated oxidative stress levels in GC. All cytokines upregulated *Tgfb1* expression, while IL-1β and IL-6 induced mild type III collagen deposition in the TC layer, suggesting early activation of fibrotic signaling. Notably, follicles with multi-layered TC structures exhibited partial resistance to TNF-α–induced oxidative stress and GC dysfunction.

**Conclusion:**

Pro-inflammatory cytokines impair early follicular development by disrupting GC and TC function through gonadotropin resistance, oxidative stress, and fibrotic signaling. These findings provide a novel insight into how chronic inflammation compromises ovarian function, and suggest that preserving the follicular microenvironment—particularly TC integrity—may be key to mitigating cytokine-induced follicular dysfunction in inflammatory reproductive disorders.

## 1 Introduction

Chronic low-grade inflammation is a persistent, subclinical inflammatory state distinct from acute inflammation caused by infection or trauma. Although often asymptomatic, it gradually disrupts tissue homeostasis through immune dysregulation, oxidative stress, and fibrotic remodeling (1). This pathological process has been linked to a wide range of systemic conditions, including metabolic syndrome, cardiovascular disease, neurodegeneration, and certain malignancies (2). Previous studies have also suggested that chronic low-grade inflammation may adversely affect female reproductive health, particularly ovarian function in infertile women (3).

The ovarian follicle—the fundamental unit of female reproduction— consists of an oocyte surrounded by granulosa cells (GC) and theca cells (TC), and interactions between these cells govern follicular development (4). Folliculogenesis from the primordial to antral follicle stage is intricately controlled by pituitary gonadotropins, particularly follicle-stimulating hormone (FSH), and ovarian factors such as steroid hormones, growth factors, and cytokines. During the transition from the preantral to early antral stage, follicles become dependent on FSH to support GC proliferation, gonadotropin receptor expression (FSH receptor [*Fshr*] and luteinizing hormone receptor [*Lhcgr*]), and steroidogenesis, particularly estradiol synthesis via aromatase (*Cyp19a1*). Failure to acquire FSH responsiveness at this critical checkpoint often results in follicular atresia (4–6).

Pro-inflammatory cytokines—including tumor necrosis factor-alpha (TNF-α), interleukin-1β (IL-1β), and interleukin-6 (IL-6)—are central mediators of chronic inflammation (7, 8). Chronic overproduction of these cytokines is known to induce cellular oxidative stress, tissue damage, and fibrotic remodeling (9, 10). TNF-α, IL-1β, and IL-6 are frequently elevated in reproductive disorders such as obesity, polycystic ovary syndrome (PCOS), endometriosis, and reproductive aging (11, 12). *In vitro* studies using monolayer GC cultures have shown that these cytokines suppress *Fshr*, *Lhcgr*, and steroidogenic enzymes while inducing reactive oxygen species (ROS), thereby compromising FSH signaling and GC function (13–18).

Despite this growing body of evidence, the majority of these findings have been derived from simple two-dimensional (2D) models that do not replicate the structural complexity of the follicle. *In vivo*, GC and TC are organized in a defined spatial arrangement, enabling dynamic paracrine and juxtacrine communication crucial for follicle growth and survival (19). To date, little is known about how pro-inflammatory cytokines influence follicular function within this intact three-dimensional (3D) context. Understanding these effects is essential for elucidating the mechanisms by which chronic inflammation impairs early folliculogenesis.

In this study, we investigated the hypothesis that pro-inflammatory cytokines disrupt follicular development by impairing GC and/or TC function during the preantral to early antral transition. Using a 3D culture model of rat preantral follicles, we evaluated the effects of TNF-α, IL-1β, and IL-6 on follicle growth, steroid production, gene expression, and tissue remodeling. Our findings demonstrate that these cytokines promoted TC proliferation, while consistently suppressing FSH-induced steroidogenesis and downregulating key differentiation markers in both GC and TC. TNF-α and IL-1β induced oxidative stress in GC. TNF-α, IL-1β, and IL-6 upregulated *Tgfb1* and modestly increased collagen III deposition, suggesting early fibrotic signaling in TC. These results provide a novel insight into how chronic inflammation impairs FSH signaling and GC/TC function during early follicular development.

## 2 Materials and methods

### 2.1 Reagents and materials

Leibovitz’s L-15 medium, α-Minimum Essential Medium (α-MEM), bovine serum albumin (BSA), HEPES buffer, insulin-transferrin-selenium-sodium pyruvate (ITS-A), L-glutamine, ascorbic acid, and CellROX Green Reagent were obtained from Thermo Fisher Scientific (Tokyo, Japan). Polyvinylpyrrolidone (PVP) was purchased from Merck (Darmstadt, Germany). Recombinant human TNF-α, IL-1β, and IL-6 were obtained from Proteintech (Rosemont, IL, USA), and recombinant human FSH was provided by the National Hormone and Peptide Program (Harbor-UCLA Medical Center, Torrance, CA, USA). Enzyme immunoassay (EIA) kits for estradiol and testosterone were purchased from Cayman Chemical (Ann Arbor, MI, USA). The Power SYBR™ Green Cells-to-CT™ Kit and Power SYBR™ Green PCR Master Mix were obtained from Ambion (Waltham, MA, USA) and Applied Biosystems (Foster City, CA, USA), respectively. Custom PCR primers were synthesized by Eurofins Genomics (Tokyo, Japan). Polyclonal anti-collagen type III (N-terminal, #22734-1-AP) antibody was obtained from Proteintech (Rosemont, IL). Alexa Fluor 488-conjugated donkey anti-rabbit IgG was purchased from Invitrogen (Waltham, MA). 4’,6-diamidino-2-phenylindole (DAPI) was obtained from Nacalai Tesque (Kyoto, Japan). The tissue-clearing reagent SCALEVIEW-S4 was acquired from FUJIFILM Wako Chemicals (Osaka, Japan).

### 2.2 Animals and follicle isolation

All animal experiments were approved by the University of Fukui Committee on Animal Care (Approval Nos. R03059, R04047, R05046, R06034) and adhered to the NIH Guide for the Care and Use of Laboratory Animals. Female Sprague Dawley rats were purchased from Sankyo Labo Service Corporation (Tokyo, Japan) and maintained under controlled environmental conditions with unrestricted access to food and water.

Large preantral follicles were mechanically isolated from excised ovaries of 14-day-old rats in L-15 medium supplemented with 1 mg/mL BSA to maintain tissue viability. Follicles were dissected from the ovarian cortex using a 28.5-gauge insulin needle (Becton Dickinson, New Jersey, USA) under a stereomicroscope (SZX16, Olympus, Tokyo, Japan), ensuring that the basement membrane and TC layer were preserved. Only structurally intact follicles with clearly distinguished GC and TC layers were selected. Follicle diameter was measured along two perpendicular axes using an IX71 inverted microscope (Olympus). Follicles with a GC layer diameter of 130–160 µm, delimited by the basement membrane, were used for culture.

Due to the preserved optical transparency of isolated preantral follicles, two distinct morphological subtypes were readily distinguishable based on the thickness of their TC layers: single-layered TC follicles, characterized by a thin TC layer, and multi-layered TC follicles, with two to three concentric TC layers (**Supplementary Figure 1**). Preliminary experiments revealed that the TC layer was ≤5 µm in single-layered follicles and ranged from 6 µm to <15 µm in multi-layered ones. Based on these criteria, follicles were categorized for experimental use as follows: those with TC layer thickness ≤5 µm were classified as single-layered TC follicles, whereas those with 6–15 µm thickness were defined as multi-layered TC follicles (**Supplementary Figure 2**). Approximately ten follicles of each subtype—single−layered and multi−layered TC follicles—were isolated from a single juvenile female rat.

To ensure model consistency, most experiments were conducted using single-layered TC follicles. However, in the final set of analyses, both follicle types were included in a direct comparison to evaluate how TC structural complexity influences follicular responses to pro-inflammatory cytokines.

### 2.3 Follicle culture conditions

Preantral follicles were individually cultured in 96-well plates (Sarstedt, Newton, NC, USA) at 37 °C under 5% CO_2_ in humidified air. The culture medium consisted of α-MEM supplemented with 2.6 mg/mL HEPES, 1 mg/mL BSA, 10 mg/mL ITS-A, 4 mg/mL L-glutamine, 5 µg/mL ascorbic acid, 100 U/mL penicillin, 100 µg/mL streptomycin, and 2% (w/v) PVP. The addition of PVP increased medium viscosity to maintain follicular spherical integrity and reduce mechanical stress.

The follicles were cultured for three days with or without 10 ng/mL of FSH and different concentrations of TNF-α, IL-1β, or IL-6 (10 or 100 ng/mL), as indicated. The FSH concentration was selected based on preliminary dose-response findings, and cytokine concentrations were based on previous *in vitro* studies investigating their effects on GC and TC (13, 15, 20–22).

Follicle growth was monitored noninvasively daily using bright-field imaging, and diameters were analyzed using cellSens Standard software (Olympus). GC volume was calculated from the distance between the basement membranes, and TC volume was calculated by subtracting GC volume from total follicle volume. Growth was quantified by comparing volume changes between days 0 and 3. Media were changed every other day, and conditioned media on day 3 were collected and stored at −20 °C for subsequent hormone assays. Data represent 12–40 follicles per group from three to six independent experiments and are expressed as mean ± standard error of the mean (SEM).

### 2.4 Steroid hormone assays

Estradiol and testosterone concentrations in day 3 culture conditioned media were quantified using commercial EIA kits, according to manufacturer protocols. Intra-assay coefficients of variation (CVs) were 10.9% for estradiol and 9.5% for testosterone; inter-assay CVs were 12.5% and 11.7%, respectively. Assay sensitivities were 10 pg/mL for estradiol and 6 pg/mL for testosterone. Results represent 4–5 follicles per group from three independent experiments and are expressed as mean ± SEM.

### 2.5 Quantitative RT-PCR

Following sonication-mediated cell lysis, total RNA extraction and cDNA synthesis from individual follicles on day 3 of culture were performed using the Power SYBR™ Green Cells-to-Ct™ Kit, in accordance with the manufacturer’s instructions. During RNA preparation, samples were treated with DNase I to eliminate potential genomic DNA contamination. Quantitative real-time PCR was performed using Power SYBR™ Green PCR Master Mix on a StepOnePlus™ Real-Time PCR System (Applied Biosystems). The thermal cycling conditions were as follows: an initial denaturation at 95 °C for 10 min, followed by 40 cycles of denaturation at 95 °C for 15 s and annealing/extension at 60 °C for 60 s.

Target genes included FSH receptor (*Fshr*), LH receptor (*Lhcgr*), cholesterol side-chain cleavage enzyme (*Cyp11a1*), 17α-hydroxylase/17,20-lyase (*Cyp17a1*), aromatase (*Cyp19a1*), anti-Müllerian hormone (*Amh*), and transforming growth factor beta 1 (*Tgfb1*). *18S rRNA* was used as the internal reference gene. Relative gene expression levels were calculated using the 2^−ΔΔCt^ method. Primer sequences are provided in **Supplementary Table 1**.

Primers for quantitative PCR were designed in-house and synthesized by Eurofins Genomics (Tokyo, Japan). Prior to experimental use, each primer pair was rigorously validated by generating standard curves to determine the slope, coefficient of determination (R²), and amplification efficiency. Only primer sets that met the following criteria were used for analysis: slope values between −3.58 and −3.10, R² values ranging from 0.950 to 0.999, and amplification efficiencies between 90% and 110%. In addition, melt curve analysis was performed after each qPCR run to confirm the presence of a single specific amplification product. Primer sets that produced primer–dimer formation or nonspecific amplification products were excluded from the study. Data represent 4–5 follicles per group from three independent experiments and are presented as mean ± SEM.

### 2.6 Immunofluorescence

To overcome the technical limitations of sectioning small follicles (diameter <200 μm), we developed a whole-mount immunofluorescence staining method. On day 3 of culture, follicles were fixed in 4% paraformaldehyde for 30 minutes, blocked with 2% horse serum, 1% BSA, and 0.1% Triton X-100, and then incubated overnight at 4 °C with rabbit anti-collagen III antibody (1:200) to detect a tissue fibrosis marker. Alexa Fluor 488-conjugated donkey anti-rabbit IgG antibody (1:1000, Invitrogen) was used as the secondary antibody, and nuclei were counterstained with DAPI. Follicles were cleared with the tissue-clearing reagent SCALEVIEW-S4 for 60 minutes and imaged with an FV1200 confocal laser scanning microscope (Olympus). This enabled visualization of the internal structure of the follicle, including the GC and TC layers, with spatial resolution. Fluorescence intensity within the TC layer was quantified using Fiji (ImageJ 2.9.0/1.53t; NIH, USA) to assess type III collagen expression. A 20−µm−wide band immediately adjacent to the outer surface of the follicular basement membrane was defined as the region of interest (ROI), and the mean fluorescence intensity within this ROI was measured. Background fluorescence was subtracted from each value to obtain corrected signal intensities. Representative images from 7–9 follicles per group are shown.

### 2.7 Detection of intracellular oxidative stress

On day 1 of culture, follicles were incubated with CellROX™ Green Reagent for 30 minutes at 37 °C. After washing, they were washed, fixed in 4% paraformaldehyde in PBS (pH 7.4) for 15 minutes, and counterstained with DAPI. The follicles were then cleared using SCALEVIEW-S4 for 60 minutes and imaged with a confocal microscope (FV1200, Olympus). To assess oxidative stress in the GC layer, fluorescence intensity of the CellROX signal was quantified using Fiji (ImageJ). ROIs were defined as the area enclosed by the follicular basement membrane excluding the oocyte, and mean fluorescence intensities were measured. Background fluorescence was subtracted from each measurement to obtain corrected values. Results represent 6–11 follicles per group from three independent experiments and are expressed as mean ± SEM.

### 2.8 Statistical analysis

Statistical analyses were performed using GraphPad Prism 8.4.3 (GraphPad Software, La Jolla, CA, USA). Two-group comparisons were assessed using Student’s t-test. For three or more groups, one-way ANOVA followed by Tukey’s *post-hoc* test was applied. Homogeneity of variance was verified prior to analysis. A P-value < 0.05 was considered statistically significant.

## 3 Results

### 3.1 Effects of pro-inflammatory cytokines on preantral follicle growth

**Figure 1:**
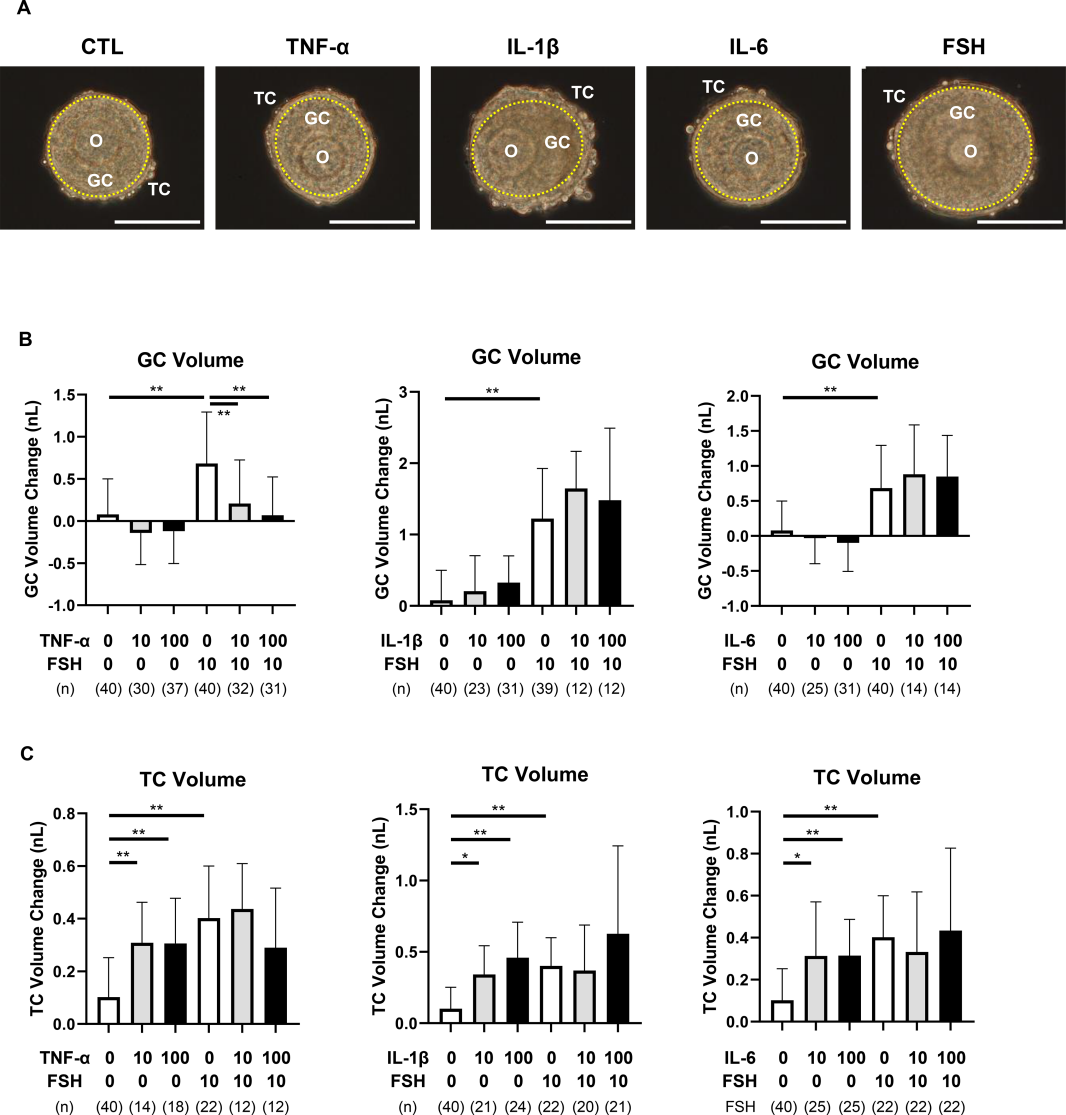
Effects of pro-inflammatory cytokines on preantral follicle growth. Rat large preantral follicles with a single TC layer (single-layered TC follicles) were cultured for 3 days with or without FSH (10 ng/mL) and different concentrations of TNF-α, IL-1β, or IL-6 (10 or 100 ng/mL). **(A)** Representative bright-field images on day 3 of culture for each treatment group: CTL (no FSH or cytokines), TNF-α (100 ng/mL), IL-1β (100 ng/mL), IL-6 (100 ng/mL), and FSH (10 ng/mL). Yellow dotted lines denote follicular basement membranes. Scale bar = 100 µm. **(B)** Volume changes of GC layer on day 3. **(C)** Volume changes of TC layer on day 3. Data are expressed as mean ± SEM from 12–40 single-layered TC follicles per group across 3–6 independent experiments. Note that TNF-α significantly inhibited FSH-induced GC proliferation, while all three cytokines independently stimulated TC proliferation. Statistical significance: *, P <.05; **, P <.01. O, oocyte; GC, granulosa cells; TC, theca cells. (n) indicates the number of follicles analyzed.

To investigate how pro-inflammatory cytokines influence *in vitro* follicular development, large preantral follicles with a single TC layer (single-layered TC follicles) were cultured for three days with or without 10 ng/mL FSH and different concentrations of TNF-α, IL-1β, or IL-6 (10 or 100 ng/mL). Representative bright-field images are shown in **Figure 1A**.

In the absence of both cytokines and FSH, GC volumes remained largely unchanged (mean change: 0.08 ± 0.42 nL). TNF-α alone had no effect on GC volume. FSH significantly promoted GC proliferation (P < 0.01), but this FSH-induced proliferation was markedly inhibited by TNF-α (P < 0.01, **Figure 1B**, **Supplementary Table 2**). In contrast, IL-1β and IL-6 had no significant impact on GC volume, irrespective of the presence of FSH.

Regarding theca cells, follicles cultured without FSH or cytokines showed minimal change in TC volume (0.10 ± 0.15 nL). However, all three cytokines—TNF-α, IL-1β, and IL-6—induced TC expansion independently of FSH (P < 0.01, **Figure 1C**, **Supplementary Table 2**). FSH alone also increased TC volume (P < 0.01), and this effect was not significantly altered by co-treatment with any of the cytokines.

### 3.2 Effects of pro-inflammatory cytokines on steroid production

**Figure 2:**
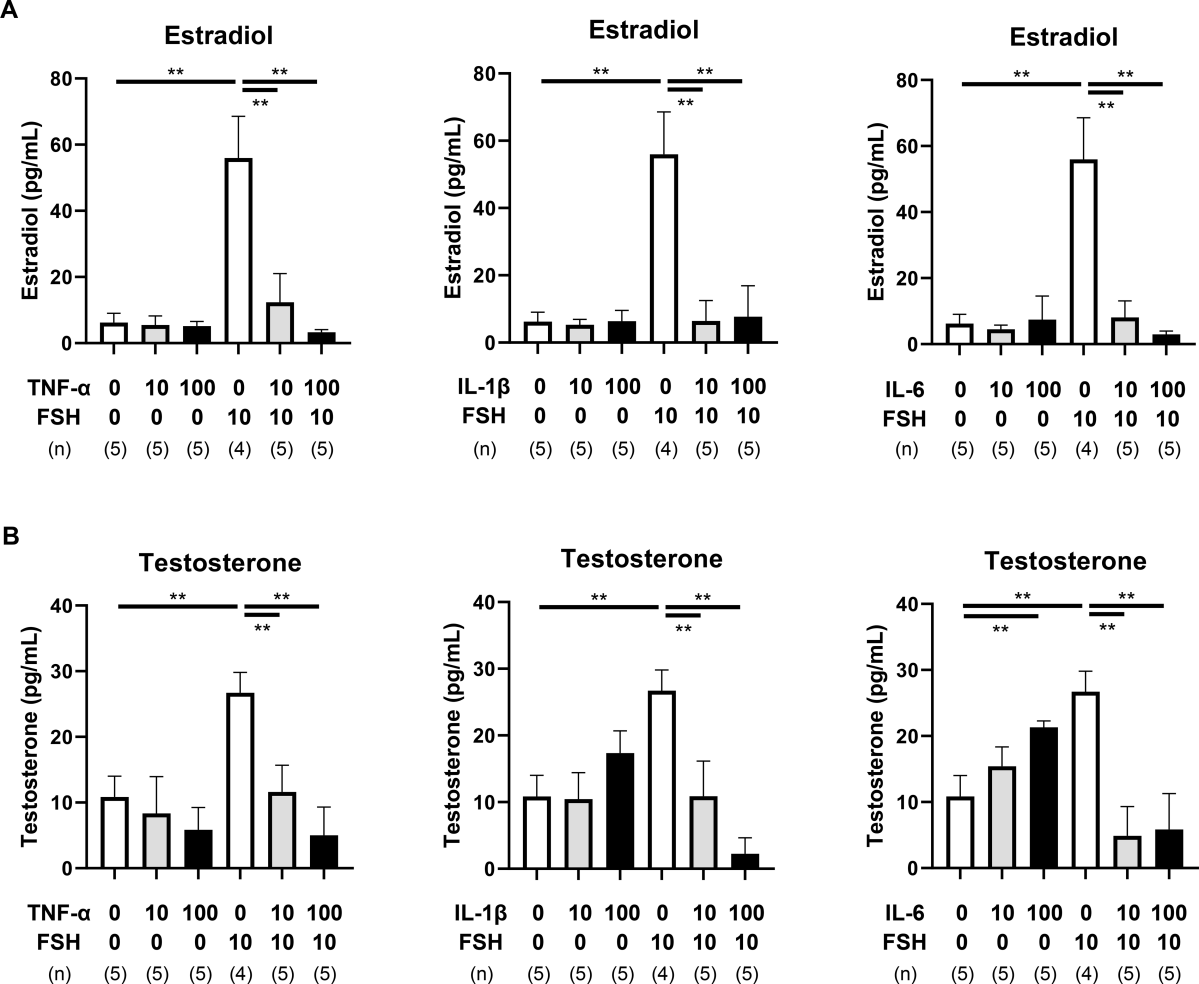
Effects of pro-inflammatory cytokines on steroid production. **(A, B)** Estradiol **(A)** and testosterone **(B)** concentrations in spent culture media on day 3. Data are presented as mean ± SEM from 4–5 follicles per group in three independent experiments. Note that TNF-α, IL-1β, and IL-6 significantly suppressed FSH-induced estradiol and testosterone secretion. Statistical significance: **, P <.01. (n) indicates the number of follicles analyzed.

Steroidogenic activity was assessed by quantifying estradiol and testosterone levels in the culture medium. In the absence of FSH, TNF-α, IL-1β, and IL-6 did not significantly affect estradiol production (**Figure 2A**, **Supplementary Table 3**). FSH treatment led to a substantial increase in estradiol levels (P < 0.01), which was significantly attenuated by all three cytokines (P < 0.01).

Testosterone levels were similarly affected. TNF-α and IL-1β had no effect on basal testosterone production, while IL-6 slightly elevated testosterone in the absence of FSH (P < 0.01, **Figure 2B**, **Supplementary Table 3**). However, the robust FSH-induced increase in testosterone was significantly suppressed by TNF-α, IL-1β, and IL-6 (P < 0.01).

### 3.3 Effects of pro-inflammatory cytokines on GC-related gene expression

**Figure 3:**
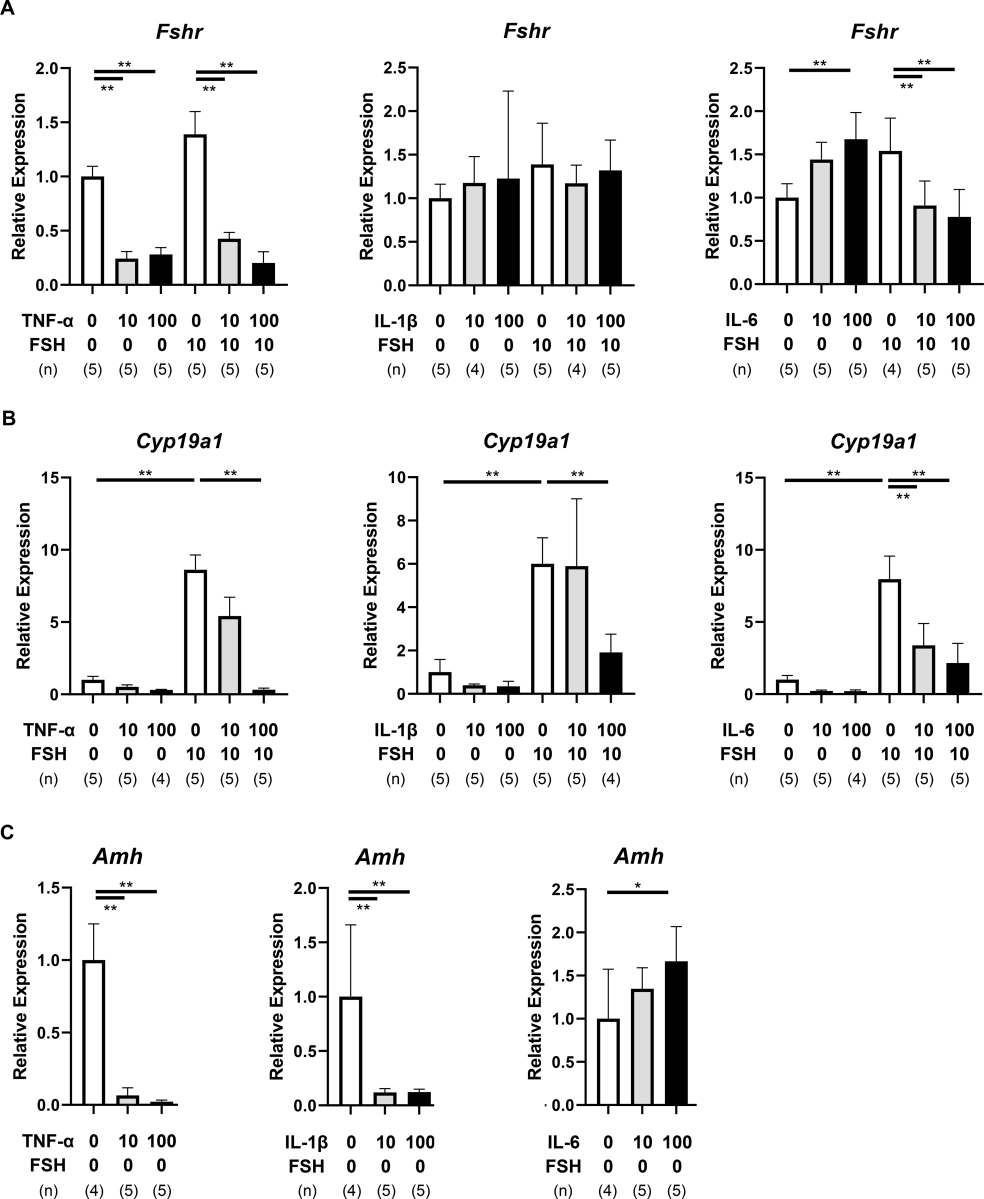
Effects of pro-inflammatory cytokines on granulosa cell-related gene expression. **(A–C)** Relative mRNA levels of *Fshr***(A)**, *Cyp19a1***(B)**, and *Amh***(C)** in the cultured single-layered TC follicles on day 3. Data represent mean ± SEM from 4–5 follicles per group from three independent experiments. Note that TNF-α significantly suppressed *Fshr* expression. All cytokines reduced FSH-induced *Cyp19a1* expression, while TNF-α and IL-1β decreased *Amh* expression. Statistical significance: *, P <.05; **, P <.01. GC, granulosa cells. (n) indicates the number of follicles analyzed.

Quantitative RT-PCR was performed to evaluate the expression of GC-related genes (*Fshr*, *Cyp19a1*, and *Amh*). TNF-α significantly downregulated *Fshr* mRNA, irrespective of FSH treatment (P < 0.01, **Figure 3A**, **Supplementary Table 4**). IL-1β had no significant effect on *Fshr*, whereas IL-6 slightly increased *Fshr* in the absence of FSH but suppressed its expression in the presence of FSH (P < 0.01).

FSH robustly upregulated *Cyp19a1* expression (P < 0.01), which was consistently suppressed by all three cytokines (P < 0.01, **Figure 3B**, **Supplementary Table 4**). For *Amh*, TNF-α and IL-1β significantly decreased expression (P < 0.01), while IL-6 induced a modest increase (P < 0.05, **Figure 3C**, **Supplementary Table 4**).

### 3.4 Effects of pro-inflammatory cytokines on TC-related gene expression

**Figure 4:**
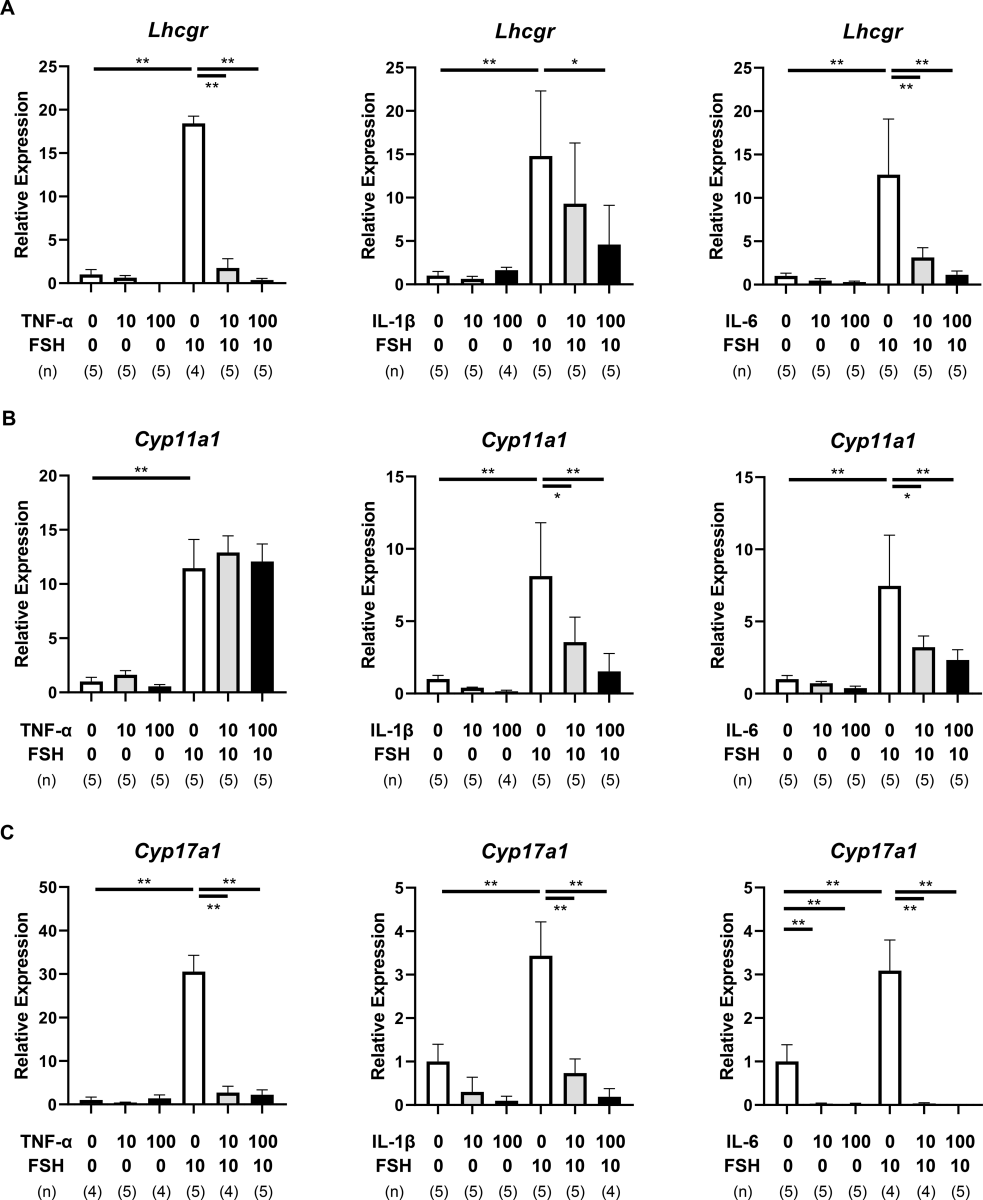
Effects of pro-inflammatory cytokines on theca cell-related gene expression. **(A–C)** mRNA expression of *Lhcgr***(A)**, *Cyp11a1***(B)**, and *Cyp17a1***(C)** in the cultured single-layered TC follicles on day 3. Data are shown as mean ± SEM from 4–5 follicles per group from three independent experiments. Note that TNF-α, IL-1β, and IL-6 significantly downregulated FSH-induced *Lhcgr* expression. IL-1β and IL-6 attenuated *Cyp11a1* expression, while all three cytokines suppressed FSH-induced *Cyp17a1* expression. Statistical significance: *, P <.05; **, P <.01. TC, theca cells. (n) indicates the number of follicles analyzed.

Expression of TC-related genes (*Lhcgr*, *Cyp11a1*, and *Cyp17a1*) was also analyzed. FSH significantly induced *Lhcgr* expression (P < 0.01), which was notably inhibited by TNF-α, IL-1β, and IL-6 (TNF-α and IL-6: P < 0.01, IL-1β: P < 0.05, **Figure 4A**, **Supplementary Table 5**). Similarly, *Cyp11a1* expression was increased by FSH and attenuated by IL-1β and IL-6, while TNF-α had no significant effect (**Figure 4B**, **Supplementary Table 5**).

In the absence of FSH, IL-6 reduced *Cyp17a1* expression (P < 0.01). FSH strongly induced *Cyp17a1* levels (P < 0.01), but this upregulation was suppressed by all three cytokines (P < 0.01, **Figure 4C**, **Supplementary Table 5**).

### 3.5 Effect of pro-inflammatory cytokines on fibrotic signaling in preantral follicles

Despite the cytokine-induced expansion of the TC layer (**Figure 1C**), all three cytokines significantly suppressed FSH-induced androgen production (**Figures 2B**, **4A–C**). To explore this discrepancy, we examined the expression of *Tgfb1*, a key mediator of fibrotic signaling.

**Figure 5:**
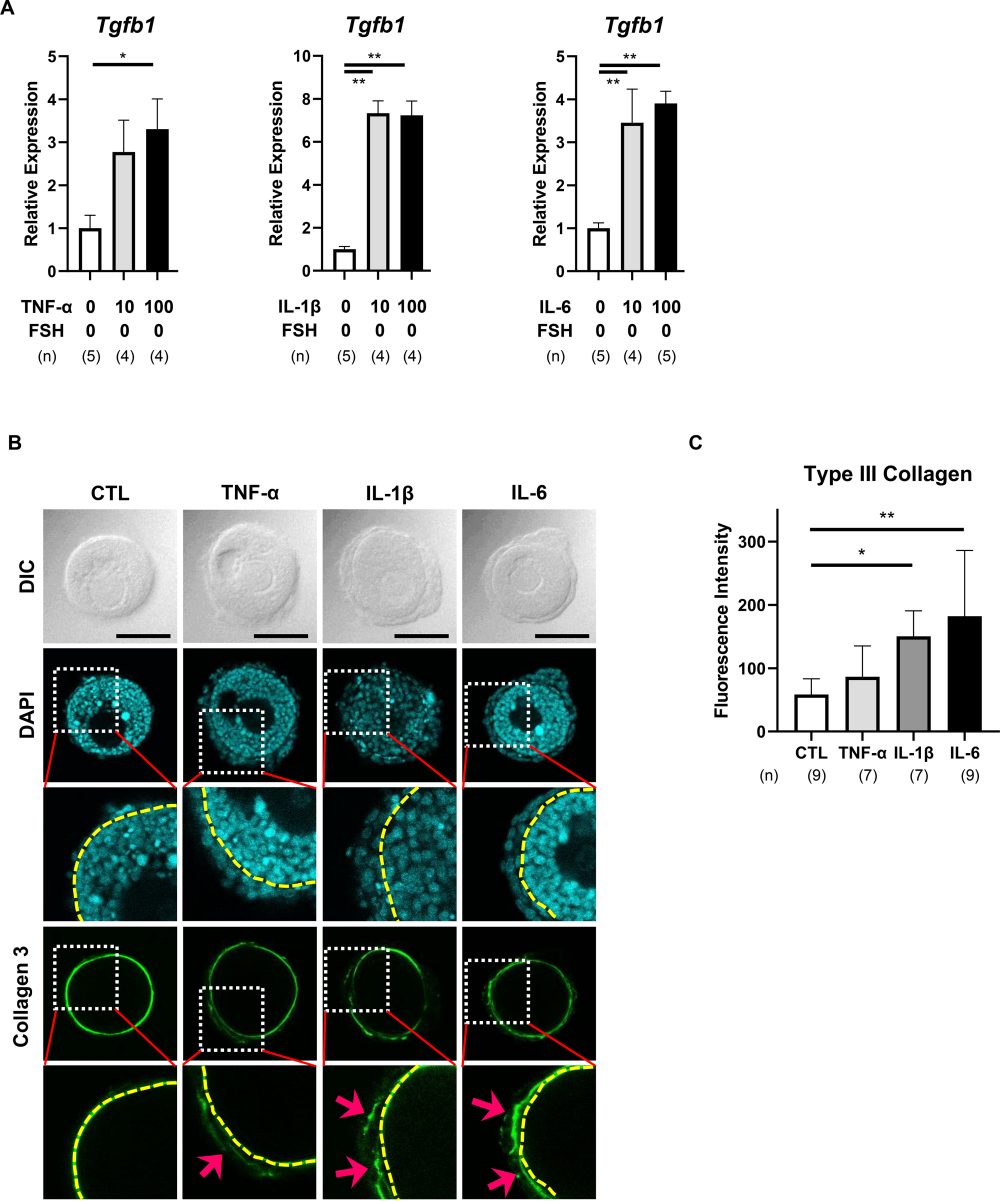
Effects of pro-inflammatory cytokines on fibrotic signaling in preantral follicles. **(A–C)***Tgfb1* mRNA expression levels in the cultured single-layered TC follicles on day 3. Data represent mean ± SEM from 4–5 follicles per group from three independent experiments. **(D)** Immunofluorescence analysis of type III collagen (a fibrosis marker) in the TC layer. Representative day 3 images from 4–5 follicles per treatment group are shown: CTL (no FSH or cytokines), TNF-α (100 ng/mL), IL-1β (100 ng/mL), and IL-6 (100 ng/mL). Yellow dotted lines indicate follicular basement membranes; pink arrows denote collagen deposition. Scale bar = 100 µm. **(E)** Quantified type III collagen deposition within the TC layer based on fluorescence intensity on day 3. Data represent mean ± SEM from 7–9 follicles per group across three independent experiments. Note that All cytokines upregulated *Tgfb1* expression, while IL-1β and IL-6 induced mild type III collagen deposition, suggesting early fibrotic signaling in the TC layer. Statistical significance: *, P <.05; **, P <.01. DIC, differential interference contrast microscopy; DAPI, 4’,6-diamidino-2-phenylindole; Collagen 3: type III collagen. (n) indicates the number of follicles analyzed.

TNF-α, IL-1β, and IL-6 markedly upregulated *Tgfb1* mRNA expression (TNF-α: P < 0.05, IL-1β and IL-6: P < 0.01, **Figure 5A**, **Supplementary Table 6**), suggesting activation of fibrosis-related pathways. Immunofluorescence staining of type III collagen further supported this hypothesis. IL-1β and IL-6 modestly increased type III collagen deposition within the TC layer (IL-1β: P < 0.05, IL-6: P < 0.01, **Figures 5B, C**, **Supplementary Table 6**), consistent with early fibrotic signaling.

### 3.6 Effect of pro-inflammatory cytokines on oxidative stress in preantral follicles

**Figure 6:**
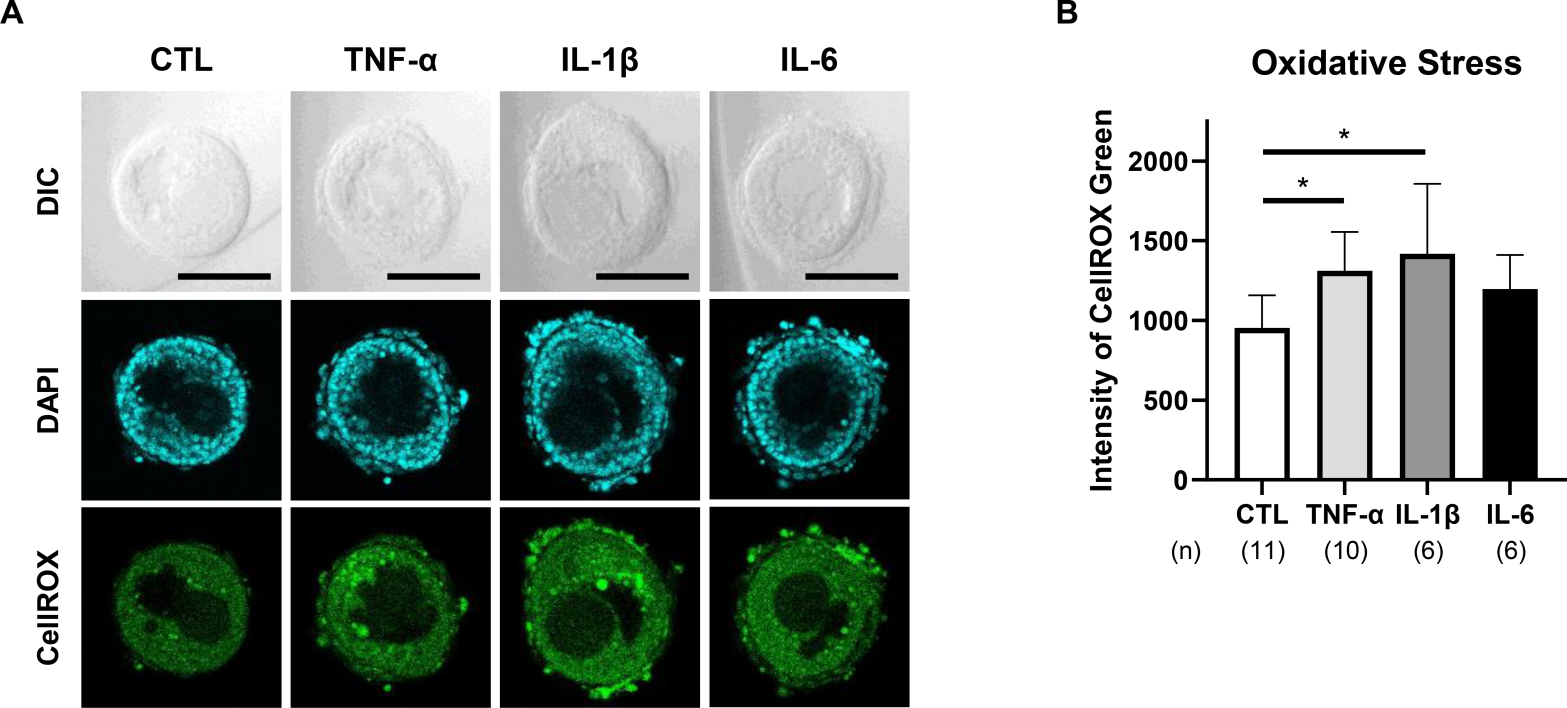
Effects of pro-inflammatory cytokines on oxidative stress in preantral follicles. Oxidative stress was assessed using CellROX Green Reagent to measure intracellular reactive oxygen species (ROS) levels in the cultured single-layered TC follicles. **(A)** Representative CellROX Green fluorescence images on day 1 for each treatment group: CTL (no FSH or cytokines), TNF-α (100 ng/mL), IL-1β (100 ng/mL), and IL-6 (100 ng/mL). Scale bar = 100 µm. **(B)** Quantified intracellular ROS levels based on fluorescence intensity on day 1. Data represent mean ± SEM from 4–5 follicles per group across three independent experiments. Note that TNF-α and IL-1β significantly increased ROS accumulation in GC, indicating cytokine-induced oxidative stress. Statistical significance: *, P <.05. DIC, differential interference contrast microscopy; DAPI, 4’,6-diamidino-2-phenylindole; CellROX, CellROX Green. (n) indicates the number of follicles analyzed.

To assess oxidative stress in preantral follicles, intracellular reactive oxygen species (ROS) were visualized using CellROX Green. Both TNF-α and IL-1β significantly elevated ROS levels in GC, as evidenced by increased fluorescence intensity (P < 0.05; **Figures 6A, B**, **Supplementary Table 7**). In contrast, IL-6 did not induce a significant rise in ROS generation in GC.

### 3.7 Protective role of multi-layered TC structure against cytokine-induced dysfunction

Two morphological subtypes of preantral follicles—single-layered and multi-layered TC follicles—were compared to determine whether TC structure influences cytokine sensitivity (**Supplementary Figures 1**, **2**).

**Figure 7:**
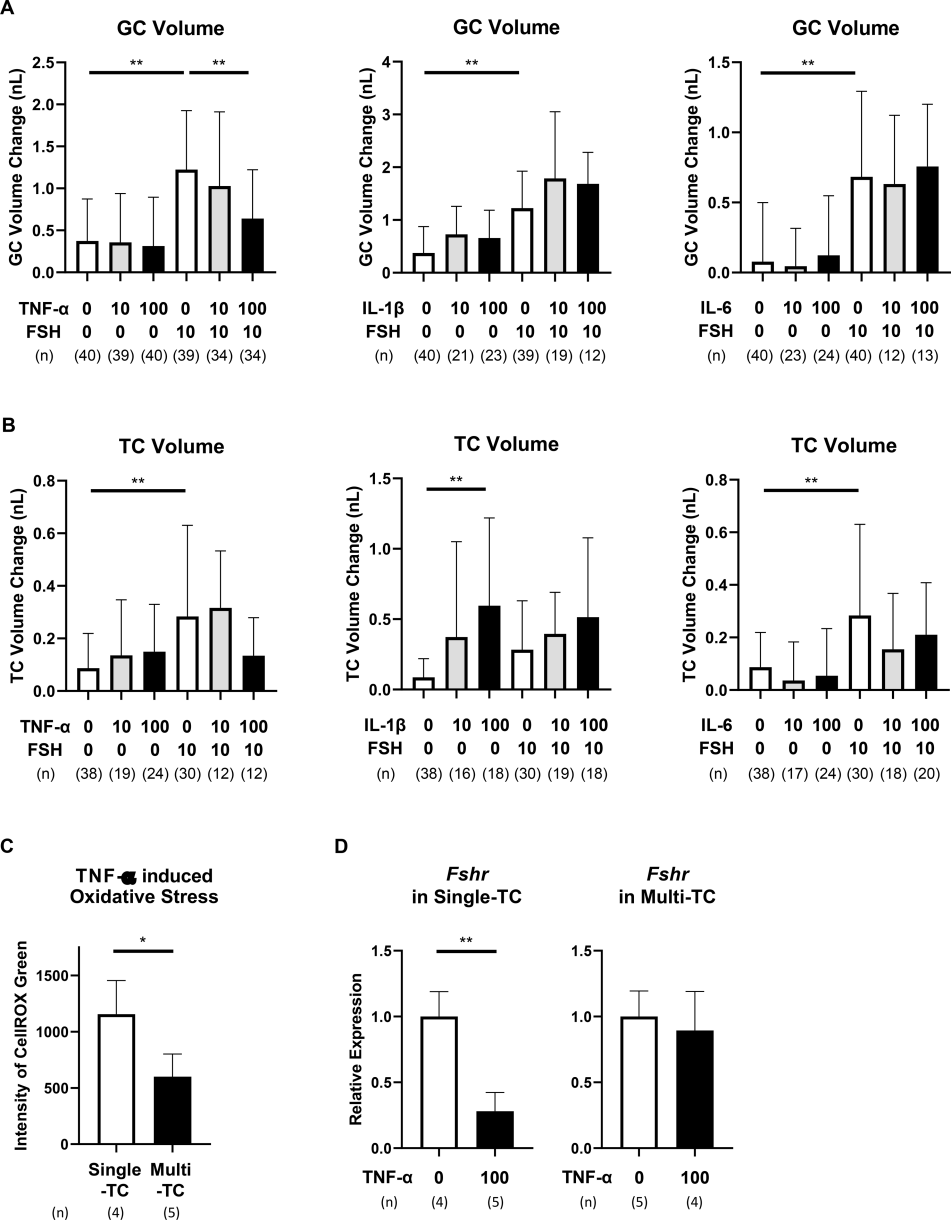
Protective role of multi-layered TC structure against cytokine-induced follicular dysfunction. Rat large preantral follicles with two to three TC layer (multi-layered TC follicles) were cultured for 3 days with or without FSH (10 ng/mL) and different concentrations of TNF-α, IL-1β, or IL-6 (10 or 100 ng/mL). **(A)** Volume changes of GC layer on day 3. **(B)** Volume changes of TC layer on day 3. Data are presented as mean ± SEM from 12–40 follicles per group across three to six experiments. **(C)** Comparison of intracellular ROS levels (single-layered vs. multi-layered TC follicles) on day 1 after TNF-α treatment. **(D)** Comparison of *Fshr* mRNA expression (single-layered vs. multi-layered TC follicles) on day 3 after TNF-α treatment. Data represent mean ± SEM from 4–5 follicles per group from three independent experiments. Note that TNF-α at 100 ng/mL suppressed GC proliferation and *Fshr* expression in single-layered TC follicles, but not in multi-layered TC follicles. ROS accumulation was also significantly lower in multi-layered TC follicles. Statistical significance: *, P <.05; **, P <.01. GC, granulosa cells; TC, theca cells; ROS, reactive oxygen species; Single-TC, single-layered TC follicles; Multi-TC, multi-layered TC follicles. (n) indicates the number of follicles analyzed.

In the absence of FSH, TNF-α, IL-1β, or IL-6 did not altere GC volume in either single-layered or multi-layered TC follicles (**Figures 1B**, **7A**). Under FSH stimulation, 100 ng/mL TNF-α suppressed GC proliferation in both subtypes. However, at a lower concentration (10 ng/mL), TNF-α significantly inhibited GC proliferation only in single-layered TC follicles, but not in multi-layered TC follicles (**Figures 1B**, **7A**, **Supplementary Table 8**). These results suggest a protective role of multi-layered TC structure against low-dose TNF-α-induced GC growth inhibition.

TNF-α and IL-6 stimulated TC proliferation in single-layered TC follicles but not in multi-layered TC follicles (**Figure 7B**, **Supplementary Table 8**). In contrast, IL-1β increased TC volume in both subtypes (P < 0.01), indicating a distinct mechanism of action.

To assess oxidative stress, intracellular ROS levels in both follicle types were measured following TNF-α treatment. Fluorescence intensity, indicative of ROS accumulation, was significantly lower in multi-layered TC follicles compared to single-layered TC follicles (P < 0.05, **Figure 7C**, **Supplementary Table 9**). Furthermore, TNF-α suppressed *Fshr* expression in single-layered TC follicles (P < 0.01) but not in multi-layered TC follicles (**Figure 7D**, **Supplementary Table 9**). These results suggest that the multi-layered TC structure confers protection against TNF-α-induced oxidative stress and GC suppression in preantral follicles.

## 4 Discussion

The present study demonstrates that cytokines known to be associated with chronic inflammation disrupts FSH-dependent folliculogenesis by impairing both GC and TC functions during early follicular development. Utilizing a physiologically relevant 3D culture system that preserves native follicular architecture and GC–TC interactions, we showed that pro-inflammatory cytokines—TNF-α, IL-1β, and IL-6—compromise FSH-induced GC proliferation and steroidogenesis, despite independently promoting TC expansion. These functional impairments were associated with downregulation of key genes involved in *Fshr*, *Cyp19a1*, *Lhcgr*, *Cyp11a1*, and *Cyp17a1*. TNF-α and IL-1β induced oxidative stress in GC. All three cytokines upregulated *Tgfb1* expression and modestly increased type III collagen deposition in TC, suggesting their involvement in early fibrotic signaling (23–25). Collectively, these changes indicate that pro-inflammatory cytokines initiate maladaptive remodeling within the follicular microenvironment, ultimately compromising follicle viability and developmental competence.

Chronic low-grade inflammation is increasingly recognized as a key contributor to female infertility, particularly in conditions such as obesity, PCOS, endometriosis, and reproductive aging (3, 26–29). These disorders are often accompanied by elevated systemic and intraovarian levels of cytokines like TNF-α, IL-1β, and IL-6. Through activation of NF-κB signaling and induction of ROS, these cytokines disrupt normal cellular signaling, induce oxidative damage, and alter tissue remodeling dynamics (18, 30). Importantly, oxidative stress may negatively regulate the expression and function of G protein–coupled receptors (GPCRs), including FSH receptor and LH receptor, via post-translational modifications and altered trafficking (31–34).

Although direct evidence within ovarian follicles remains limited, prior work suggests that elevated oxidative stress—due to aging or pathological conditions—may reduce *Fshr* expression and attenuate downstream signaling (35). One study reported that oxidative stress leads to tyrosine nitration of FSH receptor at residue Y626, which impairs its trafficking to the cell surface and results in cytoplasmic sequestration and proteasomal degradation, thereby compromising FSH responsiveness (36).

Among the three cytokines examined, TNF-α exerted the most deleterious effects on GC function. In addition to significantly suppressing FSH-induced GC proliferation and estradiol production, TNF-α markedly downregulated *Fshr* expression and increased ROS accumulation in GC. These findings align with previous reports documenting TNF-α–induced oxidative stress and apoptosis in follicular cells across multiple species (18, 30). In supplementary experiments, co-treatment with the antioxidant N-acetylcysteine (NAC) showed a modest but non-significant trend toward rescuing the TNF-α–mediated suppression of FSH-induced GC proliferation (P = 0.076, **Supplementary Figure 3**). Although this suggests a contributory role for oxidative stress in TNF-α–induced GC dysfunction, the limited effect implies that additional pathways may also be involved. Thus, TNF-α may impair FSH responsiveness in GC at least in part through redox imbalance, potentially leading to disrupted steroidogenesis, follicular atresia, and reduced ovarian function in the context of chronic inflammation.

IL-1β not only induced oxidative stress in GC but also exerted the most pronounced proliferative effect in TC, regardless of FSH stimulation or TC layering. This TC expansion was not accompanied by increased androgen production or upregulation of steroidogenic enzymes, indicating the proliferation of functionally compromised TC. Simultaneous upregulation of *Tgfb1* and modest deposition of type III collagen further implicate a fibrotic shift in the TC layer (23–25), potentially contributing to the observed reduction in steroidogenic capacity. These findings suggest that cytokine-induced TC proliferation may reflect maladaptive remodeling rather than functional compensation.

IL-6 had comparatively milder effects. Although it modestly increased *Fshr* and *Amh* expression in the absence of FSH, IL-6 still suppressed FSH-stimulated estradiol and testosterone production. It also induced *Tgfb1* expression and modest type III collagen deposition in TC, suggesting early fibrotic signaling (23–25). These findings are consistent with the pleiotropic role of IL-6 in ovarian physiology, where its effects may vary depending on concentration, duration, and local cellular context (22, 23, 37).

Importantly, follicles with multi-layered TC architecture were more resilient to TNF-α–induced oxidative stress and GC suppression than their single-layered counterparts. This suggests that increased structural complexity of the TC layer may offer a protective barrier (38, 39), possibly by limiting cytokine diffusion or buffering oxidative and inflammatory signals. These results underscore the role of follicular architecture in modulating the impact of inflammatory insults and maintaining intrafollicular homeostasis.

This study has several limitations that warrant acknowledgment. First, some analyses—such as steroid hormone assays and quantitative RT-PCR—were based on relatively small sample sizes (n = 4–5 follicles per group), due to the limited number of morphologically intact preantral follicles that could be isolated from juvenile rats.

Second, although the 3D culture system preserved native follicular architecture and GC–TC interactions, gene expression analyses were performed on whole follicle RNA. As a result, cell-type–specific responses—such as differential *Tgfb1* expression in GC versus TC—could not be resolved. Future studies incorporating single-cell RNA sequencing, spatial transcriptomics, laser-capture microdissection, or whole-mount immunostaining with Z-stack imaging would be valuable for clarifying these cell-specific responses.

Third, the cytokine concentrations used (10 and 100 ng/mL) were based on prior *in vitro* studies (13, 15, 20–22), but are likely supraphysiological. While these doses enabled detection of effects within our short-term serum-free culture model, they may not reflect physiological conditions. Although longer-term, low-dose cytokine stimulation would better reflect *in vivo* physiology, it remains technically unfeasible at present.

Finally, although the *in vitro* model provides a controlled system for dissecting cytokine effects on follicular function, it may not fully recapitulate the dynamic and systemic interactions of the *in vivo* ovarian environment. Accordingly, complementary *in vivo* studies will be essential to validate and extend these findings in a physiological context.

## 5 Conclusion

In summary, this study provides new evidence that chronic exposure to pro-inflammatory cytokines impairs early follicular development by disrupting GC and TC functions. These effects are mediated through gonadotropin resistance, oxidative stress, and fibrotic signaling within the follicular microenvironment. Although cytokines stimulated TC proliferation, this expansion did not restore steroidogenic function, indicating a shift toward a dysfunctional, fibrotic TC phenotype. Notably, follicles with multi-layered TC architecture exhibited partial protection, highlighting the importance of structural integrity in buffering inflammatory insults. Together, these findings shed light on how chronic inflammation may compromise ovarian function and suggest that targeting oxidative and fibrotic pathways could offer therapeutic avenues in inflammatory reproductive disorders.

## Data Availability

The raw data supporting the conclusions of this article will be made available by the authors, without undue reservation.
