# Supplementary figures and images for: Pro-inflammatory cytokines disrupt in vitro preantral follicle development by targeting granulosa and theca cell functions
Source: Front Endocrinol (Lausanne). 2026 Jan 16;16:1667019. doi: 10.3389/fendo.2025.1667019 (PMC12855059; doi:10.3389/fendo.2025.1667019)

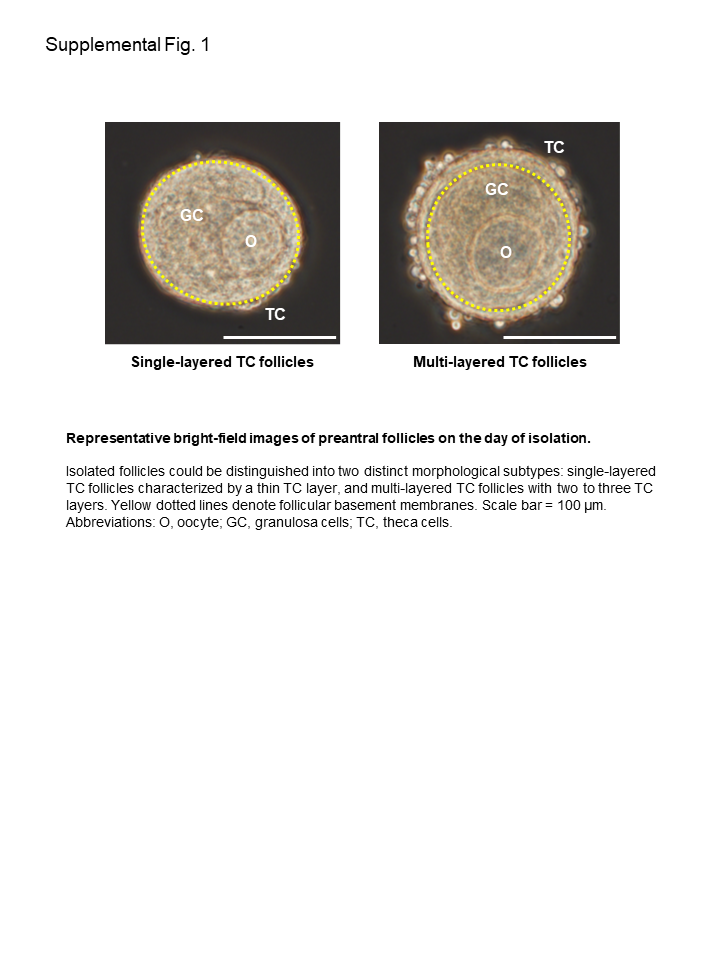

Supplement: Supplementary file 1 [file Image1.tif]

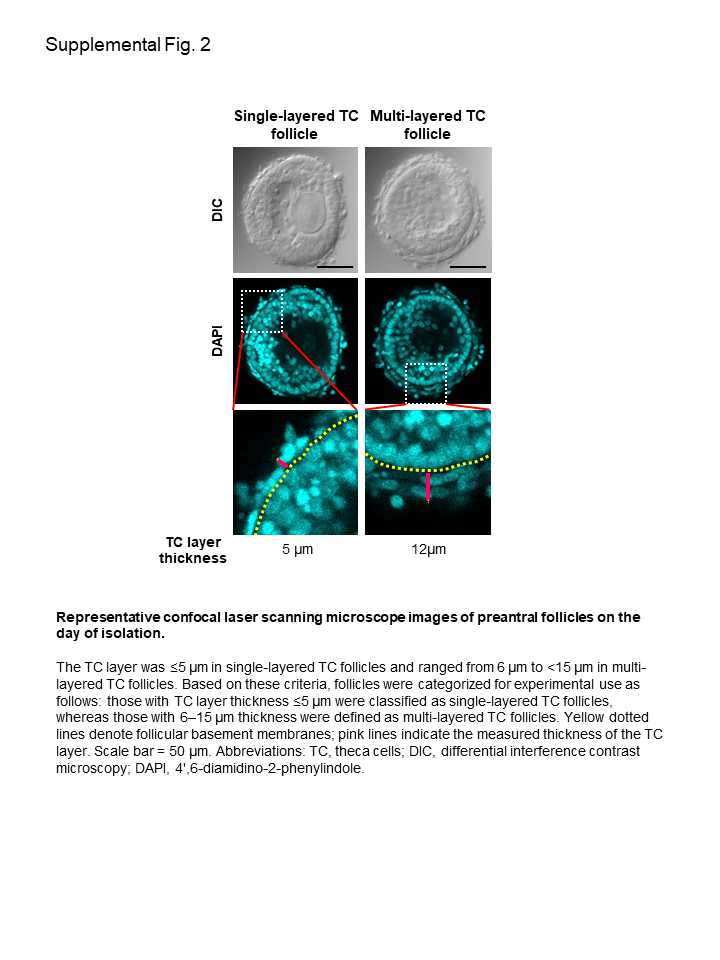

Supplement: Supplementary file 2 [file Image2.tif]

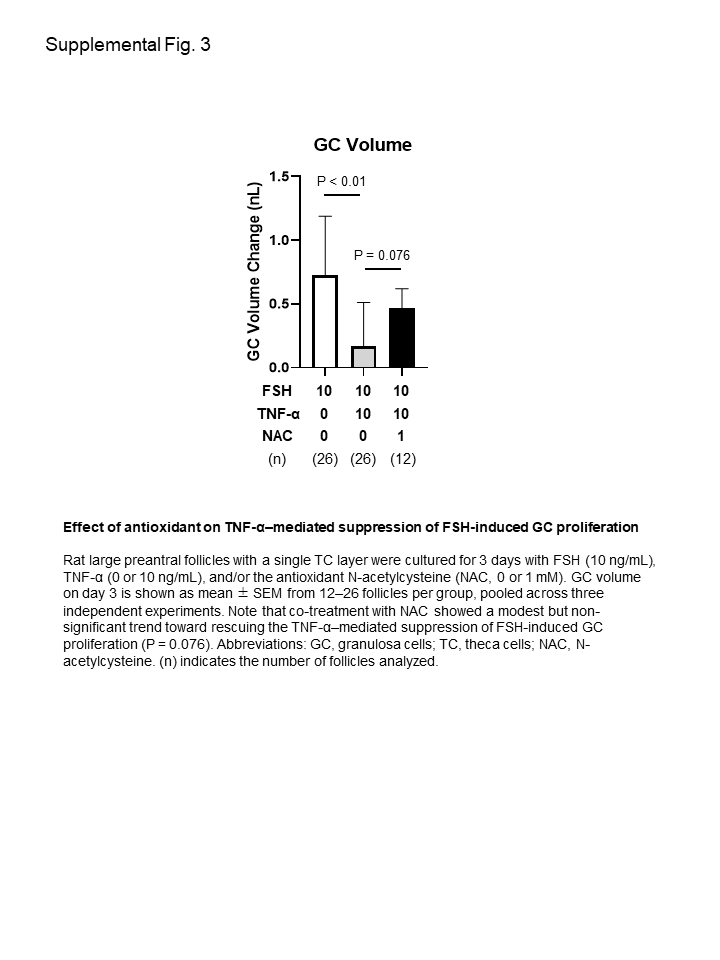

Supplement: Supplementary file 3 [file Image3.tif]

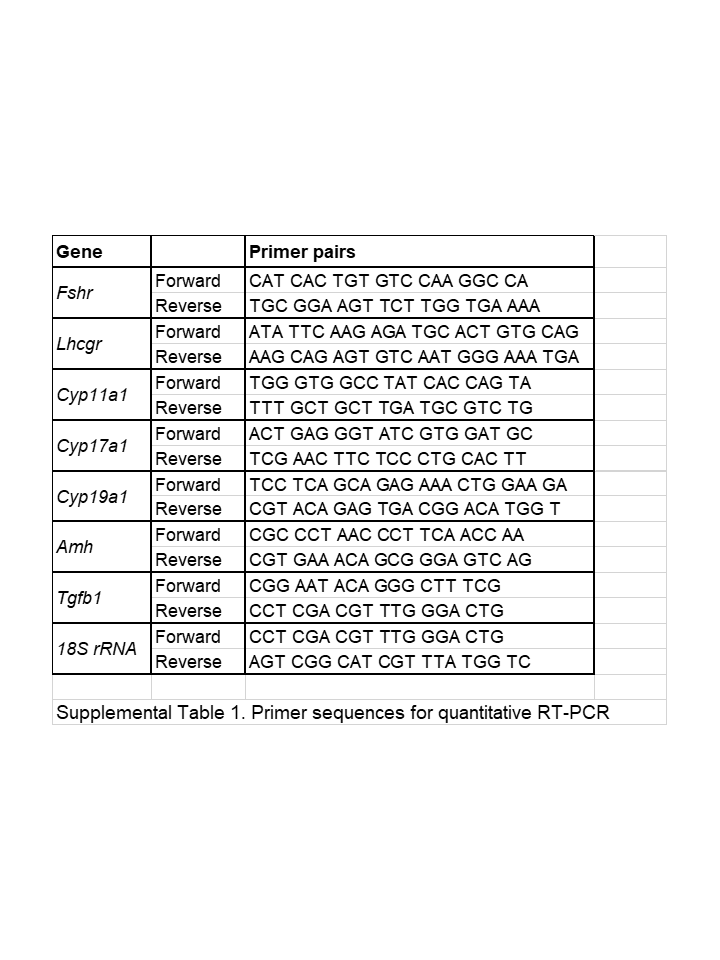

Supplement: Supplementary file 4 [file Image4.tif]

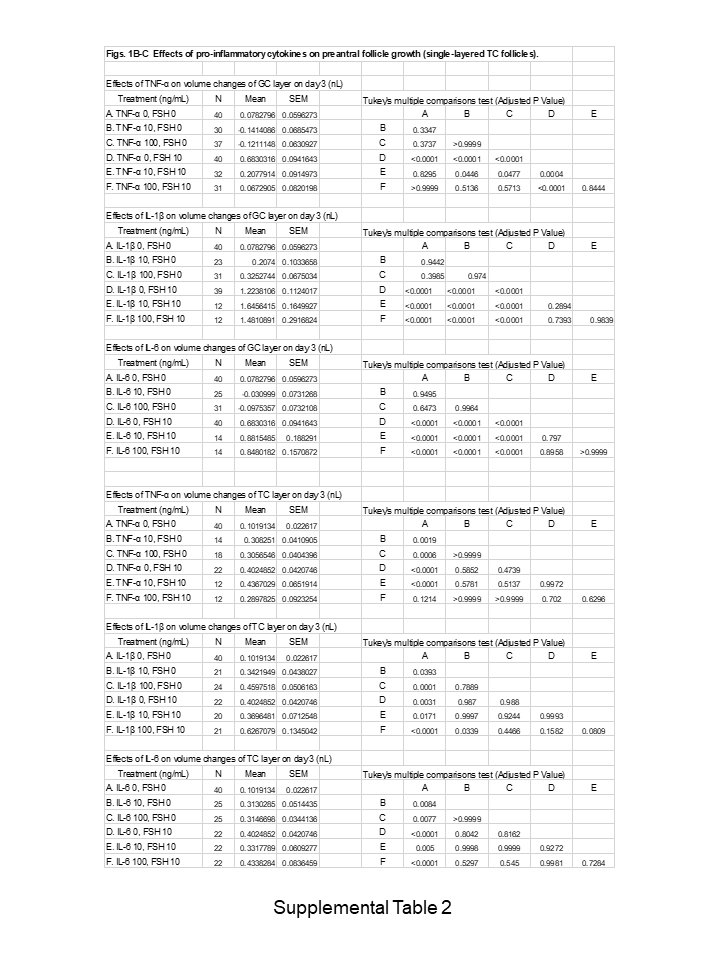

Supplement: Supplementary file 5 [file Image5.tif]

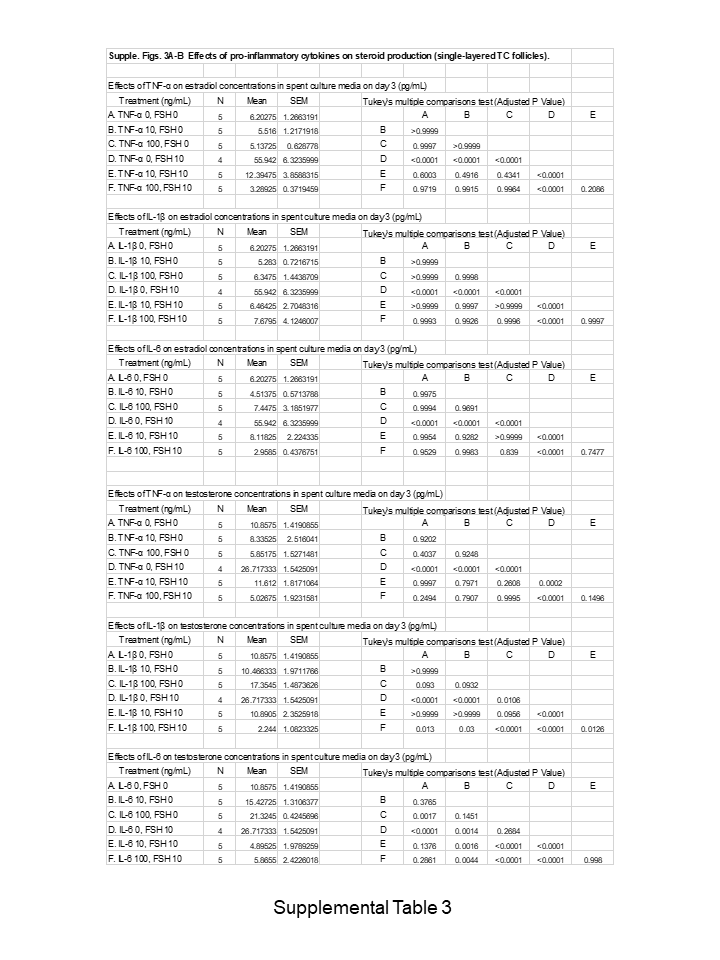

Supplement: Supplementary file 6 [file Image6.tif]

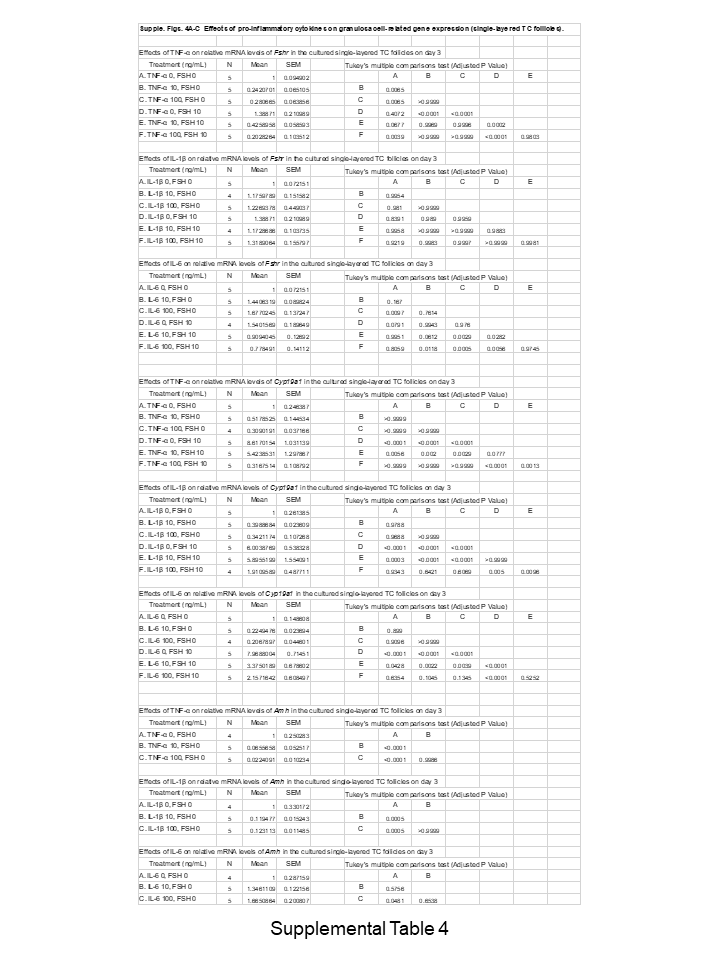

Supplement: Supplementary file 7 [file Image7.tif]

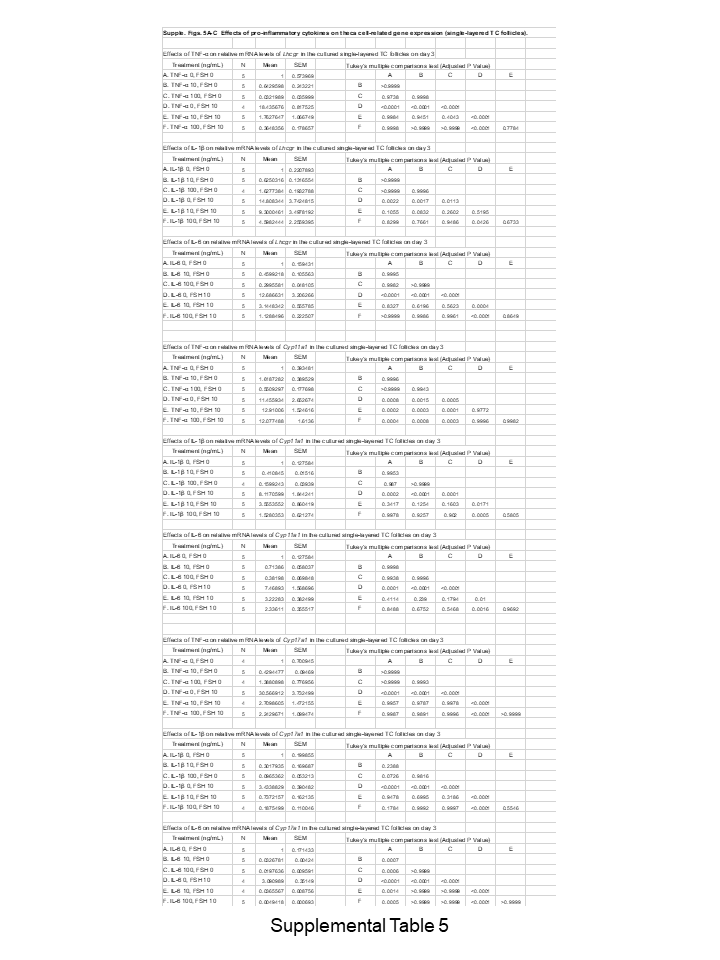

Supplement: Supplementary file 8 [file Image8.tif]

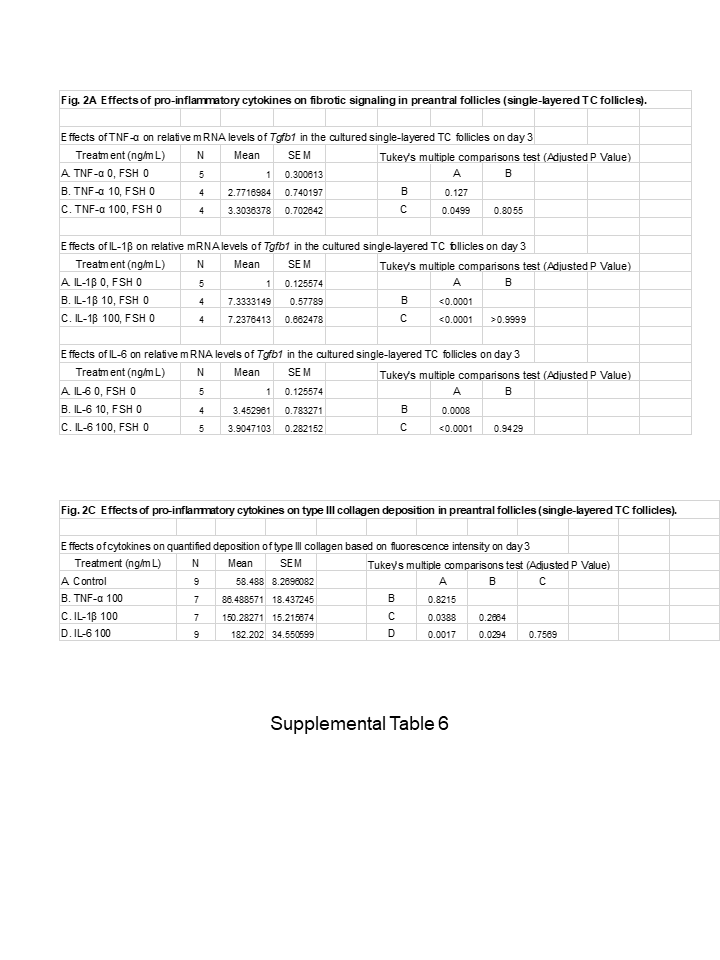

Supplement: Supplementary file 9 [file Image9.tif]

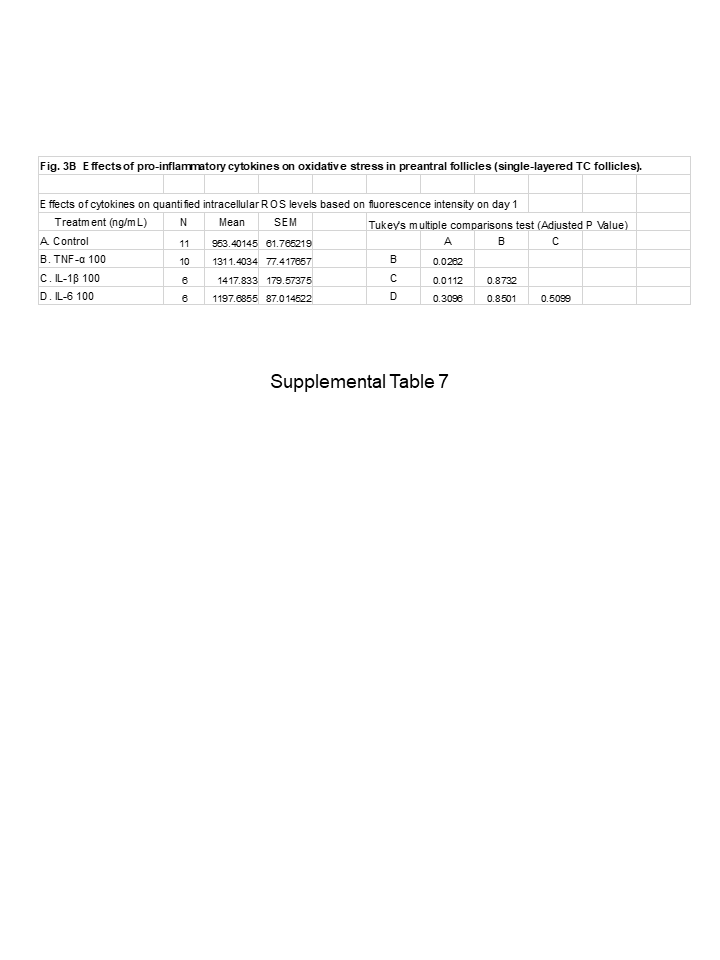

Supplement: Supplementary file 10 [file Image10.tif]

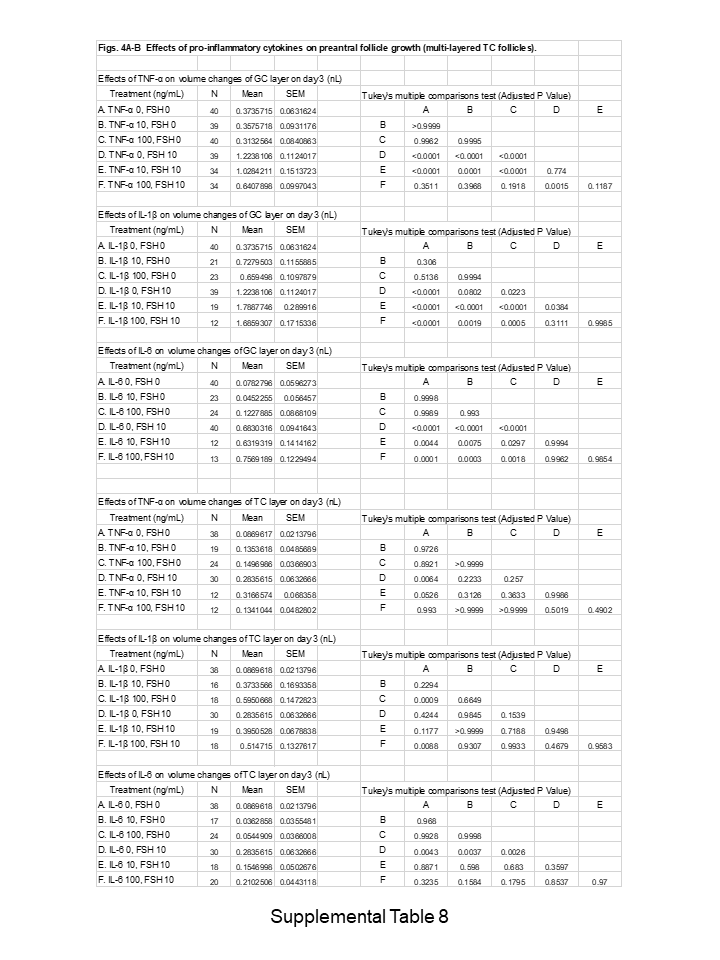

Supplement: Supplementary file 11 [file Image11.tif]

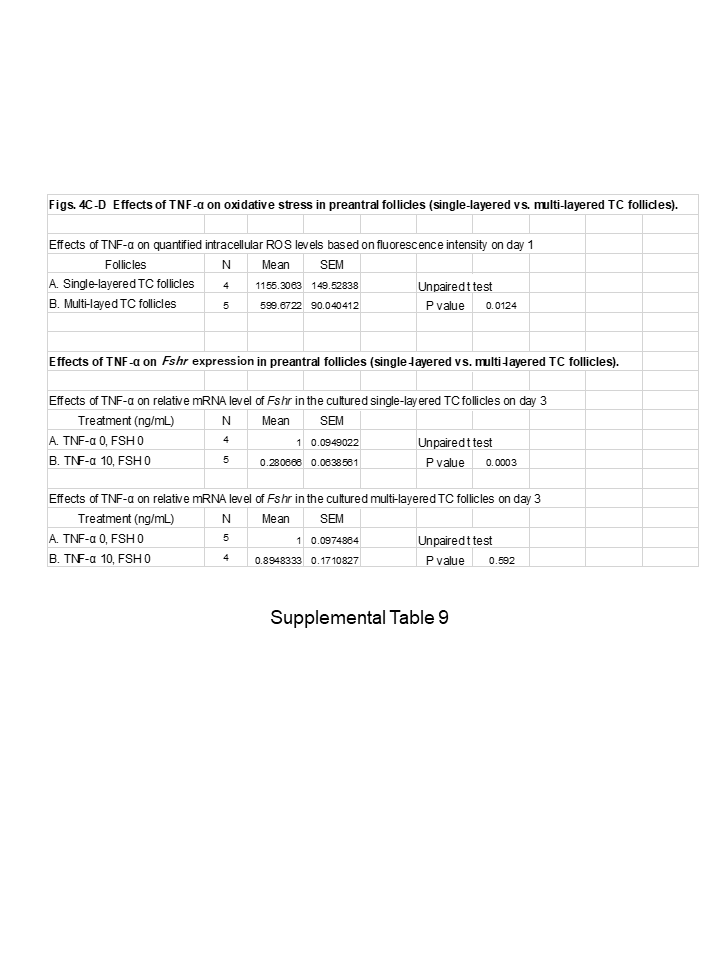

Supplement: Supplementary file 12 [file Image12.tif]
